# Daily Consumption of Golden Berry (*Physalis peruviana*) Has Been Shown to Halt the Progression of Insulin Resistance and Obesity in Obese Rats with Metabolic Syndrome

**DOI:** 10.3390/nu16030365

**Published:** 2024-01-26

**Authors:** Alberto Ángel-Martín, Fabrice Vaillant, Natalia Moreno-Castellanos

**Affiliations:** 1 Observatorio Epidemiológico de Nutrición y Enfermedades Crónicas, Nutrition School, Health Faculty, Universidad Industrial de Santander, Cra 32 # 29-31, Bucaramanga 680002, Colombia; angelmar@uis.edu.co; 2 Colombian Corporation for Agricultural Research-Agrosavia, La Selva Research Center, Kilometer 7, Vía a Las Palmas, Vereda Llanogrande, Rionegro 054048, Colombia; fabrice.vaillant@cirad.fr; 3 French Center for Agricultural Research for International Development (CIRAD), UMR Qualisud, 34398 Montpellier, France; 4 Centro de Investigación en Ciencia y Tecnología de Alimentos, Department of Basic Sciences, Medicine School, Health Faculty, Universidad Industrial de Santander, Cra 27 calle 9, Bucaramanga 680002, Colombia

**Keywords:** Golden Berry (*Physalis peruviana*), metabolic syndrome, insulin resistance, obesity, nutritional intervention

## Abstract

In a study addressing the high risk of chronic diseases in people with diabetes and obesity linked to metabolic syndrome, the impact of a Golden Berry diet was investigated using a diabetic animal model. Obese rats with diabetic characteristics were fed a diet containing five percent Golden Berry for 16 days. This study focused on various parameters including organ weights, expression of metabolic genes, and urinary biomarkers. Post-Golden Berry intake, there was a notable decrease in the body, liver, pancreas, visceral, and subcutaneous adipose tissue weights in these obese, hyperglycemic rats. In contrast, an increase in brown adipose tissue (BAT) cell mass was observed. This diet also resulted in reduced blood glucose levels and normalized plasma biochemical profiles, including cholesterol, triglycerides, LDL, and HDL levels. Additionally, it modulated specific urinary biomarkers, particularly pipe-colic acid, a primary marker for type 2 diabetes. Bioinformatics analysis linked these dietary effects to improved insulin signaling and adipogenesis. Regular consumption of Golden Berry effectively prevented insulin resistance and obesity in rats, underscoring its significant health benefits and the protective role of an antioxidant-rich diet against metabolic syndrome. These findings offer promising insights for future therapeutic strategies to manage and prevent obesity and related chronic diseases.

## 1. Introduction

Individuals with metabolic syndrome face an elevated risk of health problems like high blood glucose levels, abnormal lipid levels, high blood pressure, obesity, and insulin resistance [1]. Obesity typically stems from an excess intake of calories beyond the body’s daily requirements [2]. The World Obesity Federation defines obesity as a chronic condition that harms health, raises disease risk, and increases worldwide healthcare costs [3].

Insulin resistance occurs when cells fail to respond to insulin, causing high blood sugar levels. To counteract this, the pancreas produces extra insulin. If insulin resistance persists, it may exhaust the pancreas, increasing the risk of developing type 2 diabetes [4].

Insulin resistance is tied to factors like obesity, sedentary behavior, poor dietary choices, genetics, and environmental factors. It is also connected to health issues like cardiovascular disease and metabolic syndrome. Managing and preventing insulin resistance usually requires lifestyle adjustments, such as adopting a healthy diet and regular exercise, and sometimes, medical intervention [5].

Due to the increasing prevalence of obesity and its associated problem of insulin resistance, there is a growing interest in researching natural foods with bioactive components that may help improve these metabolic issues. The primary aim of this research is to prevent or even reverse these conditions [5].

The Golden Berry (*Physalis peruviana*), native to South America, is an exotic fruit now grown in tropical and subtropical regions. It is a small fruit, about 4 to 5 g, with a zesty outer covering. As it matures, its acidity increases while the pH decreases [6]. This fruit is a nutritional powerhouse, containing vitamins A, C, B complex, polyunsaturated fatty acids, phytosterols, and minerals [7]. This fruit is notably rich in antioxidants such as carotenoids, flavonoids, and phenolic acids, known for their role in preventing chronic diseases, including metabolic disorders. Furthermore, both in vitro and in vivo studies indicate that *Physalis peruviana* may possess anti-inflammatory, antidiabetic, and antihyperlipidemic properties [8].

Studies confirm the positive impact of Golden Berry consumption on metabolic disorders. For example, when rats on a high-fat diet were given Golden Berry, they showed reductions in body weight, total cholesterol, triglycerides, and blood glucose levels. Additionally, there was an improvement in insulin sensitivity [9]. In a similar vein, diabetic rats treated with a Golden Berry extract displayed lower blood glucose levels, improved glucose tolerance, and decreased oxidative stress markers in comparison to a control group. In sum, Golden Berry consumption has demonstrated potential in managing metabolic disorders [10]. 

Therefore, this study’s core objective is to investigate the effects of consuming Golden Berry on anatomical changes, plasma metabolites, genes, and urinary biomarkers in a rat model simulating obesity and insulin resistance.

## 2. Materials and Methods

### 2.1. Plant Material

Golden Berry “Dorada” is a variety selected by AGROSAVIA (Ref. ICA UCH-16-02) which are grown by the company Caribbean Exotic S.A.S. at the farm “La Bendición” (6.23779 latitude/75.32.45 longitude) in San Vicente Ferrer, Antioquia, Colombia [8,11].

### 2.2. Diets and Animals

This study involved 64 Wistar rats (*Rattus norvegicus*), both male and female, of the Wistar WI IOPS AF/Han type. These rats were housed in the Experimental Unit of the Industrial University of Santander. We confirmed that there were no exclusions in the study, ensuring that the sample represented the entire group of participating animals. Additionally, the assignment of the rats to the experimental control and treatment groups was conducted through a process of randomization, thereby minimizing potential biases and ensuring the reliability of the study’s findings. The sample size was meticulously calculated using a specific equation to ensure statistical validity Equation (1).

*n* = 2(*δZ*_1_ − *β* + *Z*_1_ − *α*/2)^2^*σ*^2^ (1)

(*δ* = 0.7), (*Z*_1_ = 0.842), (*β* = 0.20), (*α* = 0.05), (*σ* = 2) (measured through the standard deviation), so that their product is close to 1.401; *n* = 8. The rats were maintained under controlled conditions, with ad libitum access to food and water. The research consisted of four experimental groups: (i) Standard Diet (SD), *n* = 8; conventional diet composed of 20% protein, 16% fat, 64% carbohydrates; (ii) Standard Diet with Golden Berry (SD-GB), *n* = 8; the conventional diet was supplemented with 8% (*w*/*w*) fresh *Physalis peruviana* fruit, finely chopped into small pieces, and added to the diet; (iii) High-Fat Diet (HFD), *n* = 8; composed of 19% protein, 43% fat, 38% carbohydrates; and (iv) High-Fat Diet with Golden Berry (HFD-GB), *n* = 8; the HFD was supplemented with 8% (*w*/*w*) fresh *Physalis peruviana* fruit, finely chopped into small pieces, and added to the diet, as shown in Table 1. Abdomen circumference, body weight, and animal length were measured each week [12]. Body mass index (BMI) was calculated using the equation BMI = Weight (g)/Length (cm^2^) [13]; urine and blood samples were collected at the beginning (pre-) and end of the experiment (post-day 16). Specifically, urine samples were collected and centrifuged at 7000× *g* rpm for 15 min and filtered under pressure with a microporous syringe filter of 0.22 μm (Minisart- RC25, Sartorius., Gotinga, Alemania). After animal sacrifices, liver, pancreas, visceral adipose tissue (VAT), brown adipose tissue (BAT), and subcutaneous adipose tissue (SAT) were collected and weighed. Blood samples were centrifuged, and glycemia and lipid profile, including cholesterol, triglycerides, high-density lipoprotein (HDL), and low-density lipoprotein (LDL), were measured following the manufacturer’s protocols (Wiener Lab, Rosario, Argentina) and read at 280 nm with the spectrophotometer Synergy H1 (BioTek, Santa Clara, CA, USA) [14].

### 2.3. Gene Expression

Gene extraction and amplification was performed on SAT, and peroxisome proliferator-activated receptor gamma (PPARγ), fatty acid synthase (FasN), insulin receptor (INSR), and lipoprotein lipase (LPL) gene expression was evaluated by real-time polymerase chain reaction (qPCR) [2]. Briefly, RNA was extracted with TRIzol (Ambion, Waltham, MA, USA), followed by quantification with NanoDrop (Thermo Fisher Scientific, Waltham, MA, USA); RNA samples were purified with DNAse I treatment (Promega, Madison, Wisconsin, USA), followed by retrotranscription with M-MLV reverse transcriptase (Invitrogen, Waltham, MA, USA). Real-time qPCR amplification was carried out using primers designed with Biosearch Technologies Software (https://www.genscript.com/tools/real-time-pcr-taqman-primer-design-tool?src=google&utm_source=google&utm_medium=cpc&utm_campaign=Oligo-qPCR_Probes_Design_Tool_NA&jiraid=12194&gad_source=1&gclid=Cj0KCQiAtaOtBhCwARIsAN_x-3K2QGTD4HniIgZbNEMyTviHGdBritqIPPt8v2te-pZ0hdBJw1sS6aYaAu2pEALw_wcB accessed on 6 April 2023) (Bio-Rad, Hercules, CA, USA): Forward (F) and Reverse (R) for INSR: F: GCGGGGTGAAGACGGTCAATG, R: TGACAGGTGAAGCCCTTCATG; FasN, F: GCTGCCGTGTCCTTCTTCTACTAC, R: GGTACTTGGCCTTGGGTGTTTATAC; PPARγ, F: GAACCCAGAGTCTGCTGCTGATCTCTG, R: TCAGCGGGAAGGACTTTATGTATG and, LPL, F: GGCTCTCTGCCTGAGTTGTAGAAAG, R: TCTTGGCTCTCTCTGACCTTGTTGA. All samples were amplified in thermal cycler- CFX96 (Bio-Rad, Hercules, CA, USA), and quantitative analyses were performed with Gen 5 Software (BioTek, Winooski, VT, USA) [14].

### 2.4. Analysis UPLC/ESI-Q-Orbitrap

A UPLC-ESI-Q-Orbitrap system was used to analyze diluted urine. Chromatographic separation was performed coupled to a pre-column (130 Å, 1.7 μm particle size, 2.1 mm × 5 mm, Waters). UHPLC (Thermo Fisher Scientific, Waltham, MA, USA) was coupled to an ESI-Q/Orbitrap mass detector (Thermo Fisher Scientific, Waltham, MA, USA). The mobile phases were solvent A (water + 0.1% formic acid) and solvent B (acetonitrile + 0.1% formic acid). The elution gradient started at 0% of B. The high-resolution mass detector ESI-Q-Orbitrap system was used under a complete scan from 100 to 1000 Da in continuous mode. The column was kept at 40 °C. For untargeted metabolomics, mass spectrometry analyses were performed with a constant collision energy of 6 eV. The raw data were analyzed by progenesis QI software (https://www.waters.com/nextgen/us/en/products/informatics-and-software/mass-spectrometry-software/progenesis-qi-software/progenesis-qi.html accessed on 28 July 2023). (Waters Inc., Milford, MA, USA). Subsequently, the data drift was corrected using the Metabodrift [15]. For UPLC/ESI-Q-Orbitrap analysis, multivariate data analysis was conducted with software SIMCA Version 15.0.2 (Sartorius Stedim Data Analytical, Goettingen, Germany) in order to identify metabolites between groups with and without Golden Berry consumption (baseline) [16].

### 2.5. Discovery of Biological Association Networks

Ingenuity Pathway Analysis (IPA) software (https://analysis.ingenuity.com/pa/installer/select accessed on 25 August 2023).(Ingenuity, Redwood City, CA, USA) was used for the discovery of association networks of the urine metabolites [12]. The metabolites KEGG IDs and log2 FC (fold change) were used to identify possible interactions with biological molecules. The *p*-values were calculated using Fisher’s exact test to determine the probability of association between the metabolites in the dataset; the logarithm (*p* value) > 2 was taken as the threshold and a Z score > 2 was defined as the threshold of significant inhibition for canonical pathway, disease, and function analysis. 

### 2.6. Statistical Analysis

Statistical analysis was performed using GraphPad Prism^®^ version 8.0 (GraphPad Software, San Diego, CA, USA) and SPSS/Windows software version 15.0 (SPSS Inc., Chicago, IL, USA). All results were expressed as mean ± standard error (SEM). One-way analyses of variance (ANOVA) with Tukey’s test were applied for the analysis of more than 2 groups. In all cases, a *p*-value < 0.05 indicated a statistically significant difference between tested groups.

## 3. Results

### 3.1. Anatomical Measurements

The results in Table 2 show anatomical parameters showing how rat body weight changed after treatments with or without Golden Berry compared to their initial measurements. The post-diet (HFD-post) shows a significant increase in weight compared to its initial state (HFD-pre) in both males (*p* = 0.0014) and females (*p* = 0.0004). Among the post-treatment values, HFD-post exhibits the most significant differences compared to others, notably in males (*p* = 0.000012) and females (*p* = 0.0148) concerning SD, SD-GB (males *p* = 0.000012, females *p* = 0.00089), and HFD-GB only in males (*p* = 0.0077), as females showed no significant differences (*p* = 0.90008). Furthermore, when comparing the effects of all post-treatments, it was observed that HFD-post had significant differences compared to SD-post, but only in males (*p* = 0.0001) and not in females (*p* = 0.1440). However, HFD-post showed significant differences when compared to SD-GB-post (males *p* = 0.0020, females *p* = 0.0083) and HFD-GB-post (males *p* = 0.0277, females *p* = 0.0236).

### 3.2. Biochemical Parameters

The results presented encompass pre- and post-evaluations of various biochemical parameters under different dietary conditions, showing statistically significant differences between genders and dietary treatments. These results are divided into five categories, as shown in Table 2.

Glycemia response to diet: The HFD-post led to significantly higher levels compared to the initial HFD state (males *p* = 0.000001, females *p* = 0.000077) and compared to the SD-post (males *p* = 0.0074, females *p* = 0.0304). Interestingly, for the addition of Golden Berry (HFD-GB-post), significant differences were observed only in males (*p* < 0.000001) and females (*p* = 0.04966).

Cholesterol levels and dietary influence: Cholesterol levels spiked significantly in the HFD group across genders (males *p* < 0.000001 and females *p* < 0.000001). Compared to other diets, SD (males *p* < 0.000001, females *p* < 0.000001), SD-GB (males *p* < 0.000001, females *p* < 0.000001), and HFD-GB (males *p* < 0.000001, females *p* = 0.000003). This elevation was markedly higher, suggesting a strong impact of high-fat content on cholesterol levels. The Golden Berry inclusion in diets (both SD-GB and HFD-GB) showed an apparent moderating effect.

Triglyceride dynamics across diets: Post-HFD triglyceride levels significantly surpassed initial levels for both genders (males *p* < 0.000001, females *p* = 0.0027). In comparison to other dietary treatments, SD (males *p* = 0.00552, females *p* = 0.0010), SD-GB (males *p* < 0.000001, females *p* = 0.000002), and HFD-GB (males *p* < 0.000001, females *p* = 0.000173). This increase was the most pronounced, illustrating the impactful role of high-fat content on triglyceride levels.

LDL-cholesterol variations: Each treatment exhibited significant differences in post-treatment concentrations compared to its baseline: SD-post (males *p* = 0.0185), SD-GB-post (males *p* = 0.0042), females *p* = 0.0004), HFD-post (males *p* = 0.0350, females *p* = 0.0352), and HFD-GB-post (males *p* = 0.0018, females *p* = 0.000098). Notably, males in the SD-post group showed a unique response when compared to HFD-post (*p* = 0.0070), indicating a differential gender-specific impact of these diets on LDL levels.

HDL-cholesterol trends followed a similar pattern, showing significant differences between treatments. SD-post exhibited significant differences in comparison to its initial state (males *p* = 0.0166, females *p* = 0.0155), and SD-GB-post had significant differences compared to its baseline, but only in females (*p* = 0.0051). Differences were also observed between HFD-post and its initial state exclusively in males (*p* = 0.000083), as well as in both males and females when comparing HFD-GB-post with its initial state (males *p* = 0.000005, females *p* < 0.000001). The response of HDL levels to the diets was gender-specific and varied across different dietary compositions, particularly highlighting the role of Golden Berry.

Interconnected implications: This study reveals a complex interaction between diet composition, gender, and metabolic health. High-fat diets consistently led to adverse effects across all parameters, which were somewhat mitigated by the inclusion of Golden Berry. This indicates the potential of dietary modifications in managing diet-induced metabolic changes. Gender-specific responses further emphasize the need for personalized dietary recommendations. The cross-linking of these parameters underlines a multi-faceted approach to understanding dietary impacts on health, suggesting a broader narrative of diet and metabolic interplay.

### 3.3. Impact of Dietary Variations on Organ and Adipose Tissue Weights in Wistar Rats: The Role of Golden Berry Supplementation

The results in Table 3 show the tissue weights of rats exposed to different diets. Liver: Significant differences were observed when comparing liver weights between rats in the HFD treatment and the SD group (males *p* < 0.000001, females *p* < 0.000001), SD-GB (males *p* < 0.000001, females *p* < 0.000001), and HFD-GB (males *p* = 0.00004, females *p* < 0.000001). Additionally, SD showed significant differences in comparison to SD-GB (*p* = 0.00124) and HFD-GB (*p* = 0.000739), but only in females, while SD-GB had significant differences in relation to HFD-GB in both (males *p* = 0.00140 and females *p* < 0.000001). The liver weight, particularly in the SD-GB and HFD-GB groups, indicated a slight yet significant reduction when compared to their respective control groups. This points to a potential beneficial impact of Golden Berry supplementation in modulating liver weight, a crucial factor in metabolic health.

Pancreas: HFD resulted in higher organ weights in both males and females compared to SD (males *p* < 0.000001, females *p* = 0.000011), SD-GB (males *p* < 0.000001, females *p* = 0.000008), and HFD-GB (males *p* < 0.000001, females *p* = 0.01205). Furthermore, SD-GB had differences compared to HFD-GB (males *p* = 0.000004, females *p* = 0.003009), and in females, SD and SD-GB showed significance in relation to HFD-GB (*p* = 0.01075 and *p* = 0.003009, respectively). Regarding pancreatic weight, the variations observed across the groups were minimal, suggesting a relative stability of this organ’s mass under varying dietary conditions. This finding contributes to the understanding of the pancreas’s resilience to dietary changes.

Adipose tissues: Both VAT and SAT in males and females exhibited similar trends. HFD led to higher VAT and SAT weights in males compared to SD (VAT: *p* < 0.000001, SAT: *p* < 0.000001), SD-GB (VAT: *p* < 0.000001, SAT: *p* < 0.000001), and HFD-GB (VAT: *p* < 0.000001, SAT: *p* < 0.000001). SD also showed significant differences compared to SD-GB (VAT: *p* = 0.00066, SAT: *p* = 0.000051) and HFD-GB (VAT: *p* < 0.000001, SAT: *p* < 0.000001). Notably, when evaluating SD-GB in comparison to HFD-GB, significant differences were observed (VAT: *p* < 0.000001, SAT: *p* < 0.000001). For females, the pattern was similar to males. HFD resulted in higher VAT and SAT weights compared to SD (VAT: *p* < 0.000001, SAT: *p* < 0.000001), SD-GB (VAT: *p* < 0.000001, SAT: *p* < 0.000001), and HFD-GB (VAT: *p* = 0.002743, SAT: *p* < 0.000015). However, HFD-GB showed a reduction in adipose tissue weights compared to SD (VAT: *p* < 0.000001, SAT: *p* < 0.000001) and SD-GB (VAT: *p* < 0.000001, SAT: *p* < 0.000001), with SD-GB only displaying significant differences on SAT in comparison to SD (*p* = 0.001214). A notable observation was made in the context of visceral adipose tissue (VAT) weights. In the HFD group, a substantial increase in VAT was documented. However, in the presence of Golden Berry supplementation, this increase was significantly mitigated, implying a protective effect of this dietary component against the fat accumulation typically associated with high-fat diets.

This study also shed light on the dynamics of brown adipose tissue (BAT). A decrease in BAT weight was noted in the HFD group, whereas an increase was observed in the Golden Berry-supplemented groups. This suggests a role for Golden Berry in promoting BAT mass, a tissue known for its metabolic benefits.

Similar trends were observed in subcutaneous adipose tissue (SAT), where the HFD group exhibited increased weights, but the introduction of Golden Berry appeared to counteract this effect, especially in male rats. This finding underscores the potential of Golden Berry in regulating adipose tissue distribution and growth.

### 3.4. Comparative Analysis of Key Gene Expressions Influenced by Dietary Variations

**Figure 1:**
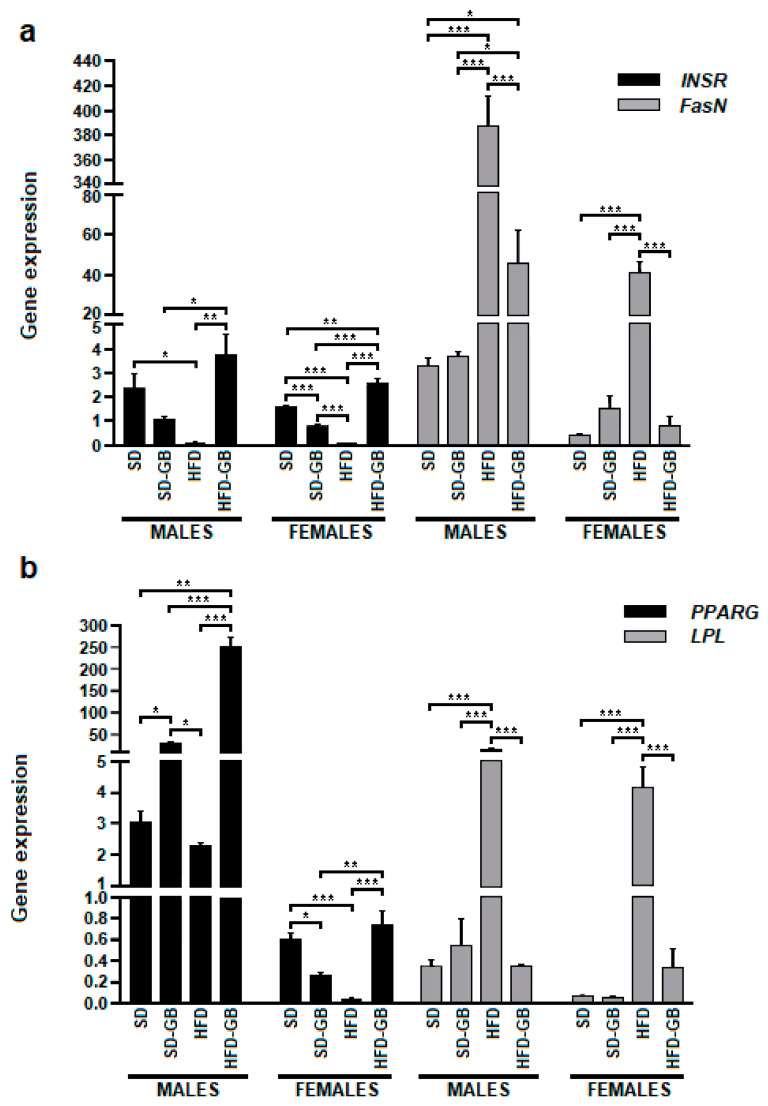
Gene expression (**a**) INSR and FasN and (**b**) PPARG and LPL in males and females in treatments with and without Golden Berry. Effects of Golden Berry supplementation on gene expression influenced by dietary variations in Wistar rats. (**a**). Gene expression of INSR and FasN: The figure shows the expression of INSR and FasN in different diets. A significant increase in INSR gene expression was observed in the HFD-GB diet compared to the HFD diet in both males (*p* = 0.0316) and females (*p* = 0.0002). Golden Berry increases INSR expression and decreases FasN expression in Wistar rats fed with HFD (*n* = 8; one-way ANOVA followed by Tukey’s multiple comparison test; ** *p* < 0.01) over a 16-week intervention period compared to rats fed with SD. (**b**). Gene expression of PPARγ and LPL: The figure illustrates the increase in PPARγ expression and the decrease in LPL, showing significant differences compared to HFD in both males (*p* = 0.04062) and females (*p* = 0.02009) and with HFD-GB. Groups are represented as (i) Standard Diet (SD), *n* = 8; (ii) Standard Diet with Golden Berry (SD-GB), *n* = 8; the conventional diet is supplemented with 8% (*w*/*w*) Golden Berry. (iii) High-Fat Diet (HFD), *n* = 8; and (iv) High-Fat Diet with Golden Berry (HFD-GB), *n* = 8; the HFD diet is supplemented with 8% (*w*/*w*) Golden Berry. * *p* < 0.05, ** *p* < 0.01, *** *p* < 0.001.

The results in Figure 1a display the gene expression of INSR and FasN. Notably, the HFD-post diet presented a significant reduction in INSR gene expression with respect to SD-post males (*p* = 0.0316) and females (*p* = 0.0002), SD-GB-post only in females (*p* = 0.01342), and HFD-GB-post males (*p* = 0.0019) and females (*p* = 0.000005). HFD-GB-post in relation to SD-GB in males (*p* = 0.0213); and in females in relation to SD (*p* = 0.003642) and SD-GB (*p* = 0.000087). As for standard diets, only females presented significant differences between SD-post and SD-GB-post (*p* = 0.02213). 

The results in Figure 1b show the expression of PPARγ for all diets tested. The highest gene expression of PPARγ was observed in the HFD-GB diet, with a significant increase compared to SD in males (*p* < 0.000001), SD-GB in both males (*p* < 0.000001) and females (*p* = 0.001193), and HFD in both males (*p* < 0.000001) and females (*p* = 0.000026). The treatment with high PPARγ expression was SD, showing significant differences compared to SD-GB in males (*p* = 0.04062) and females (*p* = 0.02009) and with respect to HFD in females (*p* = 0.00040).

LPL exhibited maximum expression in the HFD diet, which, when compared to the other diets, demonstrated high significance with respect to SD in males (*p* < 0.000001) and females (*p* = 0.000017), SD-GB in males (*p* < 0.000001) and females (*p* = 0.000028), and HFD-GB in males (*p* < 0.000001) and females (*p* = 0.00005).

These results indicate that dietary variations, especially the incorporation of Golden Berry in high-fat diets, significantly influence the expression of genes related to metabolism and insulin signaling in Wistar rats. The data suggest a notable impact of diet on metabolic gene regulation, with implications for understanding dietary influences on health.

### 3.5. Metabolic Profiling and Biomarker Discovery in Rat Urine Post-Golden Berry Consumption: An OSC-PLS-DA Approach

In the analysis of rat urine samples before and after Golden Berry consumption, significant urinary biomarkers were successfully identified with low standard deviation values in quality control pools. An OSC-PLS-DA model was applied to differentiate between urine samples of rats with and without Golden Berry consumption. This model generated a list of the most important ions based on their VIP scores, as shown in Table 4. The model’s performance was considered acceptable, with a CV-ANOVA value of 2 × 10^−5^, an R2X value of 0.46, an R2Y value of 0.99, and an RQ2 value of 0.83. Furthermore, permutation tests for cross-validation did not reveal signs of overfitting.

Unidentified compounds: The primary discriminant ions were identified as *m*/*z* 432.34 [M + H] and *m*/*z* 430.333. The first discriminant metabolites, which differ only by hydrogenation, could not be identified. The second-most discriminant metabolite had an m/z value of 380.1914 [M + H], and its fragmentation pattern corresponded to the loss of NH3 (resulting in a fragment at *m*/*z* 363.1639 with a mass loss of 17.02 amu), hydration (yielding a fragment at *m*/*z* 345.1534 with a mass loss of 18.01 amu), and glucuronidation (producing a fragment at *m*/*z* 169.11219 with a mass loss of 176.03 amu). Subsequently, phase I and II transformations led to a predominant fragment at *m*/*z* 169.11219, which displayed a fragmentation pattern consistent with geranic acid (C_10_H_16_O_2_), a compound previously observed among the flavor compounds in Golden Berry [17]. 

### 3.6. Identification and Analysis of Key Urinary Metabolites in Rats following Golden Berry Consumption

**Figure 2:**
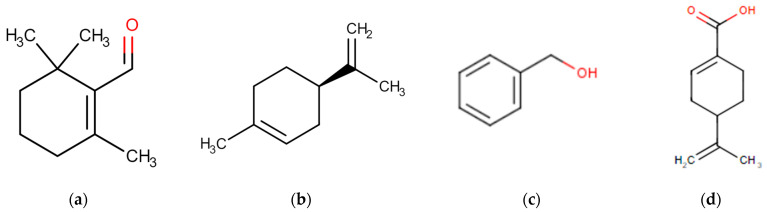
Main urinary biomarkers in rats associated with Golden Berry fruit consumption. UPLC/ESI-Q-Orbitrap metabolomic analysis of Golden Berry consumption in rats. This analysis utilized a UPLC-ESI-Q-Orbitrap system for examining diluted urine samples to assess the impact of Golden Berry (Cape Gooseberry) consumption. Chromatographic separation was achieved using a pre-column (130 Å, 1.7 μm particle size, 2.1 mm × 5 mm, Waters, Rydalmere NSW, Australia) coupled with UHPLC (Thermo Fisher Scientific, Waltham, MA, USA) and an ESI-Q/Orbitrap mass detector (Thermo Fisher Scientific, Waltham, MA, USA). The high-resolution mass detector operated in a full scan mode from 100 to 1000 Da. Untargeted metabolomic analyses were conducted with a constant collision energy of 6 eV. Data processing utilized Progenesis QI software (Waters Inc., Milford, MA, USA). Multivariate data analysis was performed with SIMCA software Version 15.0.2 (Sartorius Stedim Data Analytical, Goettingen, Germany). The key urinary metabolites identified in this study, revealing various facets of the metabolic alterations induced by Golden Berries, include β-Cyclocitral Glucuronide (**a**), Limonene Glucuronide (**b**), Benzyl Alcohol (**c**), and Perillic Acid (**d**).

In the comprehensive metabolomic analysis focusing on the impact of Golden Berry consumption in rats, several key urinary metabolites were identified, each revealing a facet of the metabolic alterations induced by this dietary intervention. Β-Cyclocitral Glucuronide, a significant monoterpene hydrocarbon in Golden Berries, emerged as a notable metabolite with its elevated presence in urine samples. This finding aligns with the high amounts of β-Cyclocitral reported in Golden Berries, underscoring its metabolic relevance, as shown in Figure 2a.

Pipecolic Acid (C_6_H_11_NO_2_), which is particularly prominent in male rats on an obesogenic diet, showed a marked decrease in urinary excretion following Golden Berry consumption, highlighting the berry’s potential role in modulating amino acid metabolism in high-fat diets. Limonene Glucuronide, tentatively identified in glucuronide form and shown in Figure 2b, and Benzyl-Alcohol Glycine, a glycosidically bound volatile in Golden Berries, were both notable for their distinct excretion patterns. These compounds underscore the metabolic processing of Golden Berry constituents, as shown in Figure 2c.

The tentative identification of D-alpha-Cyclohexylglycine, though with low confidence, adds to the diversity of metabolites influenced by Golden Berry intake. Quinoline and Perillic Acid, a significant metabolite of limonene, further illustrate the broad spectrum of metabolic changes, with Perillic Acid notably increasing in rats consuming Golden Berries. Dodecanoyl-L-Carnitine, identified with characteristic acyl-carnitine fragments, points to alterations in lipid metabolism, a finding supported by previous observations in human studies, as shown in Figure 2d.

Lastly, Acetylated Dimethylarginine, with a slight increase post-consumption, adds to the array of nitrogenous compounds affected by Golden Berry intake. Collectively, these findings provide a multifaceted view of the metabolic impact of Golden Berry consumption, revealing specific metabolites and their altered excretion patterns in rats. This intricate metabolic portrait, although requiring further validation, paves the way for a deeper understanding of the nutritional influence of Golden Berries. It is worth noting that these identifications are tentative and may require further validation and confirmation.

### 3.7. Elucidating Metabolic Networks and Biological Pathways Influenced by Golden Berry Consumption using Ingenuity Pathway Analysis

**Figure 3:**
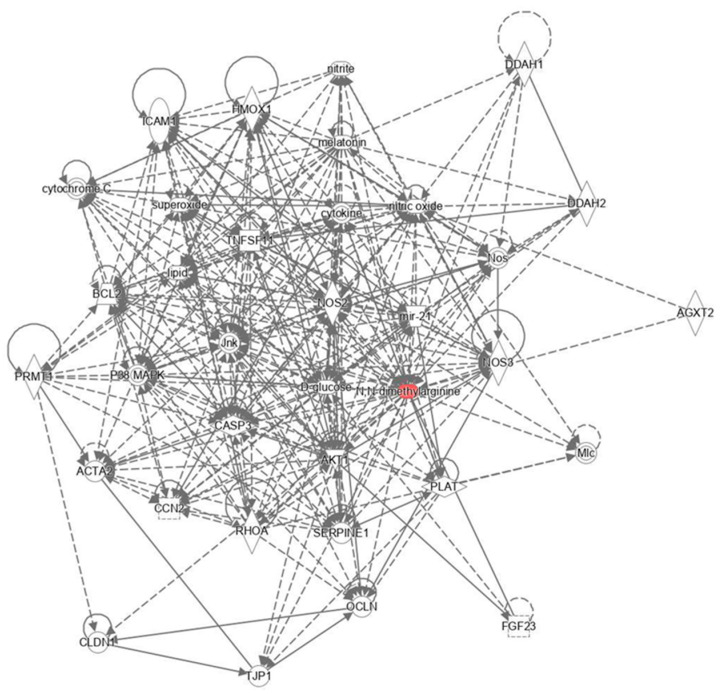
Significant biological network: impact of Golden Berry consumption. Figure 3 illustrates the significant biological network identified from the integrative association between metabolomic studies in a biological context. Utilizing the advanced analytical capabilities of Ingenuity Pathway Analysis (IPA) software, this analysis adeptly unraveled the complex metabolic association network arising from the effects of Golden Berry consumption, particularly focusing on cell death and survival, and the network of renal and urological diseases (*p*-value = 2.02 × 10^−6^). In this network representation, metabolites are depicted as nodes, with the biological relationship between two nodes indicated by a line. The colored symbols represent metabolites that not only appeared in our data but also correlate with pathways in the Ingenuity^®^ database. In contrast, transparent entries are molecules included in the Ingenuity knowledge base. Metabolites highlighted in red indicate increased levels, whereas those in green show decreased levels. Solid lines between molecules signify direct physical relationships, while dotted lines suggest indirect functional relationships.

The results in Figure 3 show the integrative association between metabolomic study in a biological context, this analysis, and the application of advanced analytical tools like the Ingenuity Pathway Analysis (IPA) software, which was adeptly utilized to dissect the intricate metabolic association network stemming from the effects of Golden Berry consumption on cell death and survival and renal and urological disease networks (*p*-value = 2.02 × 10^−6^). 

## 4. Discussion

In this groundbreaking study, we have thoroughly explored the significant impact of Golden Berry on in vivo models of Wistar rats, focusing on how this fruit alters the response to standard and high-fat diets. Our findings reveal a notable resistance to rapid weight gain and improvements in biochemical profiles when Golden Berry is incorporated into an HFD, highlighting its potential as a natural agent for weight management and metabolic control. Additionally, we have observed significant changes in the weight of key organs such as the liver and pancreas, along with alterations in gene expression related to glucose metabolism and fat synthesis. These results not only provide new insights into the benefits of Golden Berry but also open promising avenues for future research on its role in the prevention and management of metabolic disorders. In the following sections, we will detail these findings and discuss their relevance in the broader context of metabolic health and nutrition.

### 4.1. Golden Berry’s Role in Counteracting Obesity and Supporting Weight Management

An HFD is widely recognized as a contributor to obesity. However, when Golden Berry was introduced alongside the HFD, it became evident that there was a notable resistance to rapid weight gain. These results are particularly fascinating as they suggest that Golden Berry might hold promise for weight management, potentially providing valuable support for individuals striving to maintain a healthy body weight [18,19].

### 4.2. Beneficial Biochemical Effects of Golden Berry in Diet: Improved Glucose and Lipid Metabolism

Adding Golden Berry to the diet provides several health benefits, such as improved blood glucose levels and reduced insulin resistance, resulting in lower glycemia levels [20,21]. HFDs led to a significant increase in cholesterol levels, while standard diets (SD and SD-GB) contributed to substantial reductions in cholesterol levels, indicating potential heart-protective properties [22]. Golden Berry consumption in obese animals has beneficial effects. Specifically, the findings related to triglyceride levels underscore the positive impact on lipid metabolism [23,24]. Adding Golden Berry to a SD effectively controls weight gain, even in individuals with a normal body weight, by preventing rapid weight gain and the onset of chronic obesity [25,26]. Conversely, incorporating Golden Berry into the diet delays weight gain and results in a lower BMI, indicating its potential in mitigating obesity-related issues [27]. 

### 4.3. Golden Berry’s Impact on Reducing Organ Weights and Preventing Metabolic Diseases

Observations from the standard diet cohort reveal that organ weights are at the lower end of the spectrum, aligning with expectations for a diet lower in calories. The incorporation of Golden Berry into a standard diet indicates a trend towards stabilizing or slightly reducing average organ weights relative to the standard diet. This trend emphasizes Golden Berry’s potential to subtly influence organ weight, validating its application within a standard dietary framework. This benchmark establishes a crucial reference for evaluating the nuanced impacts of Golden Berry supplementation. Adding Golden Berry into the HFD diet notably reduced liver weight. This contrasts with the HFD diet, which tends to increase liver weight [28]. Excessive weight gain and high blood glucose levels can harm the liver, potentially leading to liver fibrosis [29]. The HFD regimen is starkly contrasted by its inclination to increase organ weights, notably affecting adipose tissues like VAT and SAT. This correlation starkly delineates the high-fat diet’s role in promoting body weight gain and fat accumulation. The weight gain induced by the HFD indicated a risk of liver damage. However, introducing Golden Berry into the diet significantly reduced liver weight [30]. This effect is attributed to the high levels of polyphenols, as well as vitamins A (α-carotene, β-carotene, and β-cryptoxanthin) and vitamin C found in Golden Berry [31].

Interestingly, the HFD-GB led to a decrease in pancreas size and weight, even when part of an HFD. Excessive weight gain in the pancreas, as observed in the HFD, may be linked to potential insulin resistance [32]. When it comes to adipose tissues, the data on anatomical parameters and biochemical levels strongly indicate that the HFD has a considerable impact on these measurements. This impact is most evident in the liver, pancreas, and the accumulation of VAT and SAT. However, the incorporation of Golden Berry into a standard diet indicates a trend towards stabilizing or slightly reducing average organ weights relative to the standard diet. This trend emphasizes Golden Berry’s potential to subtly influence organ weight, validating its application within a standard dietary framework, and the introduction of Golden Berry into the daily diet helps normalize these measurements [23].

The biochemical parameters in Table 2 are linked to the organ weight results in Table 3. It was previously mentioned that excessive weight gain might be associated with the rapid expansion of WAT, including visceral and subcutaneous fat [33]. The inclusion of Golden Berry in the diet led to a decrease in both organ and tissue weights and an overall improvement in biochemical profiles. Adding Golden Berry into the diet offers a preventive strategy for managing and avoiding metabolic diseases. 

The integration of Golden Berry into a high-fat diet intriguingly appears to mitigate the usual organ weight increases associated with a high-fat diet. This observation suggests Golden Berry’s efficacy in countering some of the detrimental effects of an HFD, particularly in terms of fat accumulation.

### 4.4. Influence of Golden Berry on the Reactivity of White and Brown Adipose Tissue

Adipose tissues, both VAT and SAT in males and females, showed similar trends. The HFD led to higher weights compared to the SD. However, the HFD-GB showed a reduction in adipose tissue weight compared to the SD. A notable observation was made in the context of VAT weights. A substantial increase in VAT was documented in the HFD group. However, in the presence of Golden Berry supplements, this increase was significantly mitigated, implying a protective effect of this dietary component against fat accumulation typically associated with HFDs. Adipose tissues exhibit significant weight fluctuations, highlighting their sensitivity to dietary changes and supplementation. This responsiveness is key to understanding the metabolic outcomes of different dietary patterns and interventions. 

This study notably reveals the dynamics of BAT, with a marked decrease in BAT weight in the HFD group, contrasted by an increase in groups receiving Golden Berry supplementation. This observation points to Golden Berry’s potential in augmenting BAT mass, a tissue renowned for its metabolic virtues. BAT is integral for thermogenesis and functions as an endocrine organ that influences fat and carbohydrate metabolism, which could enhance calorie burning.

This comprehensive analysis illuminates Golden Berry’s potential in regulating organ weight and attenuating fat deposition, especially in the context of an HFD. These initial findings lay a foundation for further exploration into Golden Berry’s characteristics and its prospective applications in nutrition and health. This research opens up a promising path for future inquiries into the therapeutic and nutritional potentials of Golden Berry.

### 4.5. Golden Berry’s Influence on Gene Expression in Metabolic Regulation and Health

Golden Berry consumption impacts gene expression related to metabolic health, focusing on genes involved in glucose metabolism, fat synthesis, and adipocyte differentiation. 

#### 4.5.1. INSR Gene Expression Modulation by Golden Berry

Under typical conditions, the INSR gene, integral to glucose metabolism and insulin signaling, demonstrates diminished expression in response to HFDs. This decrease in expression potentially impairs insulin signaling pathways, escalating the risk of developing metabolic disorders, notably type 2 diabetes and metabolic syndrome [34]. Crucially, the consumption of Golden Berry correlates with a noticeable upregulation of INSR expression. This upregulation signifies enhanced insulin signaling, which could serve as a protective mechanism against the onset and progression of these metabolic conditions [34]. The association of Golden Berry intake with increased INSR activity highlights its potential role in mitigating adverse metabolic effects induced by HFD patterns. This finding places Golden Berry as a significant dietary component that may offer therapeutic benefits in the context of metabolic health regulation.

#### 4.5.2. FasN Expression and the Impact of Golden Berry

FasN, an enzyme pivotal for synthesizing long-chain fatty acids and storing surplus glucose as fat within adipose tissues, typically exhibits heightened expression in response to an HFD. This overexpression is closely linked to an increased risk of obesity and associated metabolic disorders [35]. Intriguingly, the consumption of Golden Berry appears to play a modulatory role in FasN expression. The intake of Golden Berry correlates with a reduction in FasN expression, suggesting a meaningful impact on metabolic pathways. This reduction points to a potential decrease in the synthesis and accumulation of fat. Such an effect of Golden Berry consumption presents an important avenue in understanding its potential in mitigating obesity and related metabolic complications, particularly those exacerbated by HFDs.

#### 4.5.3. PPARγ Expression and Its Role in Metabolic Regulation: The Impact of Golden Berry Supplementation

PPARγ, a nuclear receptor, plays an instrumental role in adipocyte differentiation, lipid storage, and glucose metabolism. Its significant therapeutic potential, particularly in the management of type 2 diabetes and metabolic syndrome, is noteworthy. PPARγ’s involvement in these metabolic processes positions it as a key target for therapeutic strategies. In the context of dietary influence, specifically under the regime of a HFD supplemented with Golden Berry (HFD-GB), there is a notable increase in the expression of PPARγ. This heightened expression is seen as beneficial, contributing to healthier fat storage patterns and improved insulin sensitivity. Such an increase, prompted by Golden Berry intake, underscores the potential of dietary components in modulating key metabolic pathways [36,37]. 

The augmented expression of PPARγ in the HFD-GB group highlights the significant modulatory impact of Golden Berry on crucial metabolic functions. This finding is particularly promising in the context of metabolic conditions characterized by disrupted fat and glucose handling. It showcases the potential of targeted dietary interventions, such as Golden Berry supplementation, in the effective management and potential alleviation of metabolic disorders. 

#### 4.5.4. Golden Berry’s Influence on LPL Gene Expression and Metabolic Health

Lipoprotein Lipase (LPL), a gene intimately connected to lipid metabolism, demonstrates significantly elevated expression levels in animals subjected to an HFD. This heightened expression is symptomatic of metabolic imbalances, typically characterized by escalated blood glucose levels and a disrupted lipid profile. Such a profile is a common precursor to various metabolic disorders. Intriguingly, the introduction of Golden Berry into the diet manifests beneficial effects on both fat and glucose metabolism. This is evidenced by a marked reduction in LPL expression within adipose tissues. This modulation suggests a potential improvement in insulin signaling pathways and the adipogenesis process, both of which are critical components in maintaining metabolic equilibrium [38]. The consumption of Golden Berry, therefore, emerges as a promising dietary intervention. Its capacity to attenuate LPL expression, and consequently its role in reshaping metabolic profiles, positions it as a valuable nutritional tool. These findings, as indicated in this study, illuminate the potential of Golden Berry in enhancing metabolic health, particularly by mitigating the adverse effects associated with HFDs.

The modulation of key metabolic genes through Golden Berry consumption, including INSR, PPARγ, FasN, and LPL, marks a significant impact on metabolic health. These findings underscore Golden Berry’s potential as a strategic nutritional intervention to counterbalance the adverse effects associated with an HFD. This insight positions Golden Berry as a promising candidate for dietary approaches aimed at improving metabolic profiles and combating diet-induced metabolic disorders.

### 4.6. Metabolomic Insights: Golden Berry’s Effect on Metabolic Biomarkers and Potential in Diabetes Management

The results in Table 4, which show a metabolomic analysis of rat urine before and after Golden Berry consumption, reveal several significant metabolites.

The discovery of Geranic Acid in Golden Berry, denoted by a specific fragment at *m*/*z* 169.11219, stands out as a notable aspect of the fruit’s metabolomic profile. As a primary compound within Golden Berry, the unique ion pattern of Geranic Acid underscores its significance in the berry’s metabolic processes. This finding enhances our understanding of the metabolomic intricacies of Golden Berry. Additionally, the identification of *p*-menth-4(8)-ene-1,2-diol in Golden Berry’s volatile fraction, characterized by fragments at *m*/*z* 329.158 and *m*/*z* 153.127, further emphasizes the complexity and richness of its metabolomic composition. These specific compounds contribute to the distinctive characteristics of Golden Berry, revealing a multifaceted metabolic profile that warrants further exploration [39]. This qualitative assessment not only highlights the diverse nature of Golden Berry’s metabolites but also suggests a broader implication of these compounds in the fruit’s potential health benefits. The presence of such unique components like Geranic Acid and *p*-menth-4(8)-ene-1,2-diol illustrates the intricate biochemistry of Golden Berry, contributing to its growing recognition in nutritional and health-related research.

A key discovery in the nutritional analysis of Golden Berry is the high concentration of glycine, an amino acid identified by a specific fragment at 109.0680. Glycine stands out for its abundant presence in Golden Berry, distinguishing this fruit as a significant source of this crucial nutrient. Glycine plays an essential role in glycine conjugation, a vital metabolic process. This process is instrumental in the excretion of aromatic acids, compounds that are crucial in various bodily functions. Glycine’s involvement in this mechanism underlines its importance in maintaining metabolic health and facilitating detoxification processes in the body [40]. This insight into Golden Berry’s glycine content adds to the understanding of its nutritional value, emphasizing the fruit’s potential benefits in supporting metabolic functions and overall health. This finding underscores the importance of considering the comprehensive nutritional profile of foods in dietary planning and health management. 

Perillic Acid (C_10_H_14_O_2_), identified by an ion at *m*/*z* 167.1067, emerges as a significant metabolite associated with the consumption of Golden Berry. As a primary circulating metabolite derived from limonene, which is abundantly present in the aroma of Golden Berry, the notable increase in its excretion in rat urine underscores the fruit’s substantial impact on limonene metabolism. This observation, as illustrated in Figure 2, not only accentuates the metabolic processing of limonene in the body but also highlights the unique biochemical footprint of Golden Berry consumption. The presence and increased excretion of Perillic Acid signify the metabolic transformation of limonene, pointing towards an enhanced biotransformation process influenced by the ingestion of Golden Berry. This finding enriches our understanding of the metabolic pathways activated by the fruit and underscores its potential role in modulating specific metabolite profiles within the body.

#### 4.6.1. Evaluation of Metabolic Impact through Acylcarnitines Post-Golden Berry Consumption

The metabolic influence of Golden Berry consumption extends to lipid metabolism, as evidenced by the increase in acylcarnitines both in rat and human urine. This is highlighted by the identification of Dodecanoyl-L-carnitine through an ion at *m*/*z* 374.35 [M + H], which exhibited characteristic acyl-carnitine fragments. The observed elevation in acylcarnitines following Golden Berry intake suggests a significant modulation of lipid metabolism [8,41]. This particular increase in acylcarnitines, such as Dodecanoyl-L-carnitine, not only indicates an enhanced lipid metabolism but also hints at a broader metabolic reconfiguration. Acylcarnitines, known for their role in transporting fatty acids into mitochondria for beta-oxidation, are crucial markers in understanding the metabolic shifts induced by dietary changes. The fact that these changes are consistent across both rat and human models further underscores the potential of Golden Berry as a significant dietary component influencing lipid metabolism. In light of these findings, Golden Berry emerges as a dietary factor with the potential to influence lipid metabolism pathways significantly. This observation opens up new perspectives on the metabolic impacts of Golden Berry consumption, suggesting a role beyond basic nutrition and pointing towards its potential utility in managing metabolic health.

#### 4.6.2. Acetylated Dimethylarginine and Golden Berry’s Metabolic Impact 

The provisional identification of Acetylated Dimethylarginine, marked by an ion at 245.15 *m*/*z* [M + H], and the observed subtle elevation in its excretion after consuming Golden Berry introduce an additional facet to our comprehension of the berry’s metabolic effects. This nuanced observation suggests that Golden Berry intake might influence the body’s arginine metabolism, potentially impacting nitric oxide synthesis pathways. Acetylated Dimethylarginine, a known inhibitor of nitric oxide synthase, plays a critical role in regulating vascular function and endothelial health. The slight increase in its excretion could imply a modulatory effect of Golden Berry on this important metabolic pathway, shedding light on its broader implications for cardiovascular health and metabolic processes. This finding, while preliminary, underscores the complexity of Golden Berry’s influence on metabolic health and warrants further investigation to fully elucidate its impact on arginine metabolism and related physiological functions.

#### 4.6.3. Golden Berry’s Influence on Metabolic Pathways Linked to Diabetes

The observed reduction in 2-Indolecarboxylic Acid, as indicated by the ion at *m*/*z* 146.06, posits an intriguing aspect of Golden Berry’s impact on metabolic processes. This particular change points towards Golden Berry’s potential role in modulating metabolic pathways that are closely associated with diabetes. The presence of 2-Indolecarboxylic Acid is a notable marker in metabolic studies, especially in the context of glucose regulation and insulin sensitivity. The specific reduction of this compound, as evidenced by the ion at *m*/*z* 146.06, could be indicative of biochemical shifts brought about by Golden Berry consumption. This reduction aligns with the potential regulatory effects of Golden Berry on pathways implicated in diabetes pathogenesis. It suggests a modulation of metabolic processes that could have implications for managing blood sugar levels and improving insulin response.

The data point to Golden Berry’s capacity to influence key metabolic pathways, reinforcing its potential as a dietary component beneficial for metabolic health, particularly in the context of diabetes prevention and management. The qualitative evidence of Golden Berry’s influence on 2-Indolecarboxylic Acid levels provides a foundation for further exploratory studies. These findings could pave the way for more in-depth research into how Golden Berry consumption might contribute to the modulation of diabetes-related metabolic pathways.

Overall, these findings collectively provide significant insights into the metabolic changes induced by Golden Berry consumption. The array of identified metabolites, ranging from amino acids to volatile compounds, underscores the fruit’s potential in influencing metabolic processes, particularly those relevant to diabetes management. This integrated metabolomic profile offers a valuable perspective on the biochemical pathways affected by Golden Berry, paving the way for further exploration of its therapeutic potential.

### 4.7. Golden Berry’s Multifaceted Impact on Metabolic Health and the Potential for Personalized Nutritional Therapy

The integration of metabolomic data into a biological context, as illustrated in Figure 3, employs advanced analytical methods. This analysis uncovers the metabolic associations resulting from Golden Berry consumption and reveals profound insights into the connections among specific discriminatory metabolites. 

Golden Berry consumption activates pathways related to endothelial function and cardiovascular health. This encompasses molecules associated with oxidative stress response, inflammation, apoptosis, and endothelial function, suggesting potential cardiovascular health benefits [42].

The antioxidants in Golden Berry, along with compounds like NAC, may ameliorate inflammation and oxidative stress, thereby enhancing insulin sensitivity. This effect could safeguard insulin-producing cells and promote effective glucose regulation, offering therapeutic potential for conditions like type 2 diabetes mellitus [43,44].

Golden Berry may bolster the body’s defense against oxidative stress by elevating NAC levels, contributing to the neutralization of reactive oxygen species [45].

While increased ADMA levels might initially seem detrimental due to their inhibitory effect on nitric oxide production, in contexts like type 2 diabetes where oxidative stress is high, this increase might be protective. Golden Berry’s antioxidant properties could help counterbalance these effects, potentially protecting against cardiovascular complications [46].

Regulators such as AMPK and mTOR signaling pathways may orchestrate a comprehensive metabolic response to Golden Berry consumption. This response entails enhanced antioxidant defenses, inflammation modulation, and complex effects on cell survival and apoptosis [42,47].

Golden Berry’s consumption results in complex metabolic effects with implications for cardiovascular health, insulin sensitivity, and antioxidant defense. This positions it as a significant subject for nutrition and potential therapeutic applications. Moreover, this integrated analysis presents opportunities for personalized nutritional strategies tailored to an individual’s genetic makeup [48].

The dietary recommendation suggests that a 60 kg individual should consume 85 g of Golden Berry daily as part of a balanced nutrition regimen. In contrast, findings from animal studies indicate that to achieve the same metabolic outcomes observed in the pathological rat model of obesity, the same individual would need to ingest a substantial 4.8 kg of fresh Golden Berry. Such a significant discrepancy points to the raw fruit’s comparatively lower concentration of the active ingredients found in its pharmaceutical counterpart. This difference further accentuates the prospective advantages of formulating pharmaceutical-grade concentrates or supplements derived from Golden Berry. Future studies building on this foundation can further demystify the therapeutic potentials and health benefits of this important fruit.

### 4.8. Expanding the Horizon: New Insights into Golden Berry’s Role in Metabolic Regulation

While existing research has laid the groundwork for understanding Golden Berry’s impact on metabolism, our study makes considerable advances in expanding this knowledge. We distinguish our research in several key areas, offering novel insights into the multifaceted role of Golden Berry in metabolic regulation. Our study delves into the intricate mechanisms through which Golden Berry exerts its metabolic benefits. By identifying specific enzymes and pathways influenced by Golden Berry consumption, we provide a more detailed and comprehensive understanding of its role in metabolic processes. This in-depth mechanistic exploration helps to clarify the precise biochemical interactions involved, shedding light on the therapeutic potential of Golden Berry

Venturing beyond established impacts, we investigated Golden Berry’s effects on additional metabolic disorders or parameters not previously studied. Our findings reveal that Golden Berry extends its positive influence to newly studied aspects of metabolic health. This expansion of its known benefits underscores the fruit’s versatility and broadens the scope of its potential applications in treating and managing diverse metabolic conditions.

Distinguishing our research from prior studies, we conducted a comparative analysis between Golden Berry and other treatments or interventions used in metabolic disorder management. This comparative approach provides invaluable insights into how Golden Berry stacks up against other options, highlighting its efficacy and potential advantages in a therapeutic context.

An essential component of our study is the examination of the long-term effects and safety profile of Golden Berry consumption, particularly in the context of metabolic disorders. This aspect of our research addresses a gap in existing studies, offering a vital perspective on the sustainability and safety of Golden Berry as a long-term dietary inclusion for individuals with metabolic concerns.

## 5. Conclusions

Golden Berry’s emergence as a potent agent in reversing obesity and insulin resistance pathologies in animal models marks a significant breakthrough in metabolic health research. This comprehensive study underscores its profound influence on critical metabolic parameters, including the regulation of glucose, cholesterol, triglycerides, and both LDL and HDL levels. 

Beyond its nutritional value, Golden Berry demonstrates a remarkable ability to modulate gene expression and influence urinary excretion patterns of essential metabolites. This study offers pioneering insights into the complex mechanisms governing insulin signaling, lipid metabolism enzymes, lipid mobilization, and the adipogenesis process. The wide-ranging impact of Golden Berry on these metabolic pathways highlights its potential as a key player in addressing chronic health conditions, especially metabolic syndrome.

Golden Berry supplementation’s effect on reducing organ weights and its influence on the reactivity of both white and brown adipose tissue in Wistar rats further suggests its role in obesity management and weight control.

Our findings confirm the significant impact of Golden Berry on crucial metabolic regulatory genes like INSR, FasN, PPARγ, and LPL. This indicates its ability to modulate key biological pathways, enhancing its therapeutic potential.

By identifying significant urinary metabolites and elucidating metabolic networks affected by Golden Berry consumption, our study contributes valuable insights into its potential role in diabetes management. The evaluation of metabolic impact through acylcarnitines and other biomarkers post-Golden Berry consumption further underscores its effect on pathways linked to diabetes.

This research not only significantly enriches our current knowledge but also opens new horizons for personalized nutrition and therapeutic strategies, representing a trailblazing advancement in nutrigenomics and metabolomics and integrating anatomical measurements, biochemical parameters, and the effects of dietary variations. By unraveling the multifaceted effects of superfoods like Golden Berry, we move towards a future where nutrition transcends conventional guidelines, evolving into a personalized science that caters to individual metabolic needs and responses. The comprehensive impact of Golden Berry on metabolic health underscores its potential role in personalized nutritional therapy, offering effective, targeted approaches to health and wellness.

## Figures and Tables

**Table 1:** The composition of rat diets.

Diet Type	Protein (%)	Fat (%)	Carbohydrates (%)	Kcal/g
**SD**	20	16	64	4.8
**HFD**	19	43	38	5.37
**GB**	Supplemented with 8% (*w*/*w*) fresh Golden Berry fruit, finely chopped into small pieces, and added to the diet.			

Standard Diet (SD), High-Fat Diet (HFD), Semi-purified diets were selected for their ability to provide consistent and precise nutrient intake. These diets were composed of refined ingredients, each sourced specifically for its nutritional content (proteins, fats, carbohydrates). Two commercial products were utilized for vivarium use. Stringent monitoring of raw material batches was conducted to ensure these Conventional Nutrition formulas were devoid of any biological, environmental, or physical contaminants. The consumption of Golden Berry (GB) was conducted using fresh fruit, which was carefully chopped into pieces to facilitate its intake by the animals.

**Table 2:** Pre and post results of anatomical and biochemical parameters in treatments with and without Golden Berry applied to the in vivo model of male and female Wistar rats.

SEX	Anatomical Parameters	SD		SD-GB		HFD		HFD-GB	
		Pre	Post	Pre	Post	Pre	Post	Pre	Post
**MALES**	**Weight**	370.3 ± 17.89	397.2 ± 17.49	395 ± 20.88	414.4 ± 16.5	418.8 ± 1.448	527 ± 18.45 ^b,d,e^	422.2 ± 9.42	457.1 ± 9.44 ^f^
	**BMI**	0.218 ± 0.006	0.221 ± 0.014	0.206 ± 0.002	0.195 ± 0.006	0.218 ± 0.003	0.343 ± 0.035 ^b,d,e^	0.258 ± 0.005	0.258 ± 0.003 ^f^
**FEMALES**	**Weight**	286 ± 9.77	311.8 ± 8.46	271.1 ± 3.79	296.1 ± 1.10	279.3 ± 0.54	342.1 ± 8.53 ^d,e^	281.4 ± 10.41	310.5 ± 2.53 ^f,g^
	**BMI**	0.191 ± 0.012	0.191 ± 0.012	0.182 ± 0.002	0.181 ± 0.004	0.184 ± 0.002	0.217 ± 0.007 ^b,d,e^	0.212 ± 0.009	0.219 ± 0.003 ^b,d^
**SEX**	**Biochemical parameters**	**SD**		**SD-GB**		**HFD**		**HFD-GB**	
		**Pre**	**Post**	**Pre**	**Post**	**Pre**	**Post**	**Pre**	**Post**
**MALES**	**GLY**	177 ± 959	209 ± 10.17	176.5 ± 11.96	208.3 ± 10.38	131 ± 9.64	253.3 ± 4.63 ^b,d^	129.5 ± 7.03	99.25 ± 7.98 ^b,d,f^
	**CHO**	31 ± 3.97	40.14 ± 5.87	31.17 ± 4.21	38.67 ± 5.68	29.33 ± 3.48	119 ± 9.41 ^b,d,e^	27.5 ± 2.78	26 ± 2 ^f^
	**TG**	53.23 ± 3.97	66 ± 1.41	58.67 ± 2.33	29.67 ± 1.45 ^b^	39.61 ± 4.82	91.4 ± 9.51 ^b,d,e^	58.67 ± 3.93	35 ± 1.85 ^b,f^
	**LDL**	198.5 ± 38.78	284.8 ± 25.08 ^a^	77.6 ± 0.76	236.4 ± 16.48 ^c^	91.6 ± 10.29	168 ± 21.3 ^b,e^	78.93 ± 1.07	254.5 ± 11.01 ^g^
	**HDL**	240.1 ± 39.88	346.9 ± 21.12 ^a^	200.3 ± 39.71	278.7 ± 15.01	132.8 ± 6.55	302.5 ± 12.19 ^e^	122.9 ± 2.23	275.1 ± 7.52 ^g^
**FEMALES**	**GLY**	190.2 ± 2.65	176 ± 10.97	171.6 ± 15.96	186.7 ± 16.22	144.9 ± 14.04	231 ± 10.73 ^b,e^	165.3 ± 17.45	191 ± 13.61 ^f^
	**CHO**	26.33 ± 1.20	27.67 ± 0.88	24.5 ± 1.26	25.67 ± 1.76	17.67 ± 1.45	53 ± 3.79 ^b,d,e^	27 ± 1.16	32.75 ± 1.38 ^f^
	**TG**	72.75 ± 1.49	72.2 ± 9.48	65.8 ± 5.01	35.33 ± 1.67 ^b,d^	74.25 ± 2.39	125.3 ± 8.29 ^b,d,e^	88.2 ± 10.61	57.33 ± 3.33 ^f^
	**LDL**	262.9 ± 3.37	260.1 ± 8.60	117.1 ± 43.51	279.6 ± 5.03 ^c^	101.1 ± 4.02	209.3 ± 10.28 ^e^	33.33 ± 4.35	215.7 ± 4.90 ^g^
	**HDL**	215.7 ± 35.49	340 ± 15.49 ^a^	159.8 ± 32.39	304 ± 7.78 ^c^	240.1 ± 56.74	312.4 ± 9.52 ^d^	76.29 ± 1.47	284.9 ± 7.71 ^g^

SD: Standard Diet; SD-GB: Standard Diet with Golden Berry; HFD: High-Fat Diet; HFD-GB: High-Fat Diet with Golden Berry; pre: before; and post: after treatment. BMI: body mass index; GLY: glycemia; CHO: cholesterol; TG: triglycerides; LDL: (low-density lipoprotein) cholesterol; HDL: (high-density lipoprotein) cholesterol. ^a^ vs. SD pre; ^b^ vs. SD post; ^c^ vs. SD-GB pre; ^d^ vs. SD-GB pre; ^e^ vs. HDF pre; ^f^ vs. HDF post; ^g^ vs. HDF-GB.

**Table 3:** Mean and standard error of relative organ weights in different treatment groups with and without Golden Berry in an in vivo model of Wistar rats (g/kg body weight).

SEX	Organ	SD (g/kg)	SD-GB (g/kg)	HFD (g/kg)	HFD-GB (g/kg)
**MALES**	**Lv**	29.25 ± 4.80	25.14 ± 3.93	30.61 ± 16.77 ^a,b^	28.59 ± 12.63 ^a,b,c^
	**Pc**	1.72 ± 0.27	1.51 ± 0.70	1.63 ± 0.99	1.63 ± 0.61
	**VAT**	15.70 ± 0.0569	8.9 ± 0.1263	39.0 ± 0.9886 ^a,b^	24.19 ± 0.3004 ^a,b,c^
	**BAT**	4.11 ± 0.0546	4.76 ± 0.0320	0.93 ± 0.0356 ^a,b^	2.19 ± 0.0121 ^a,b,c^
	**SAT**	18.32 ± 0.0832	10.76 ± 0.1515	40.13 ± 0.2659 ^a,b^	32.46 ± 0.6124 ^a,b,c^
**FEMALES**	**Lv**	26.49 ± 8.97	25.24 ± 99.72	31.54 ± 17.05 ^a,b^	29.04 ± 15.41 ^a^
	**Pc**	1.64 ± 0.50	1.55 ± 16.27	2.37 ± 8.49 ^a^	2.11 ± 1.69 ^a^
	**VAT**	13.67 ± 0.0815	12.71 ± 0.0498	53.46 ± 0.917 ^a,b^	45.15 ± 0.9088 ^a,b,c^
	**BAT**	3.70 ± 0.0105	5.66 ± 0.0210	1.39 ± 0.0132 ^a,b^	3.02 ± 0.0119 ^a,b,c^
	**SAT**	17.33 ± 0.0717	13.30 ± 0.1447	49.80 ± 0.4652 ^a^	44.89 ± 0.6132 ^a^

Values are presented as mean ± standard error in grams per kilogram of body weight. SD: Standard Diet; SD-GB: Standard Diet with Golden Berry; HFD: High-Fat Diet; HFD-GB: High-Fat Diet with Golden Berry. Lv: liver; Pc: pancreas. VAT: visceral adipose tissue; BAT: brown adipose tissue; SAT: subcutaneous adipose tissue. ^a^ vs. SD; ^b^ vs. SD-GB; ^c^ vs. HFD.

**Table 4:** Main urinary biomarkers of Golden Berry consumption in rats.

Obs *m*/*z* [M + H]	Rt (min)	FORMULA	VIP Score	Z	Tentative Identification	HMDB	Error (ppm)	MS2 Measured (%peak)	*p*-Value	Average Relative Intensity	
			TU vs. TS						B/A	Before	After GC
432.3470	12.55	C_27_H_45_NO_3_	3.29	1	Unknown			98.0965 (100); 81.07 (10)	9.5 × 10^−3^	<10	5,008,467
430.3314	12.46		2.95	1	Unknown			98.0965 (100); 81.07 (10)		<10	300,000
380.1914	11.73	C_10_H_16_O_2_-glu-H_2_O-NH_3_	2.62	1	Geranic acid	0303972	2	345.1534 (100); 169.1219 (60) 151.1114 (30)	3.2 × 10^−4^	<10	188
364.1963	13.24	C_10_H_16_O-glu-H_2_O-NH_3_	2.01	1	*p*-menth-4(8)-ene-1,2-diol	0035706	3	329.1594 (20); 153.1270 (100) 135.1165 (100)	1.3 × 10^−2^	<10	190,928
184.0968	11.59	C_7_H_8_O-Glcycine	2.62	1	Benzyl-alcohol glycine	0003119		109.0640 (80) 81.07 (100)	31 × 10^−2^	<10	77,000
158.1175	12.44	C_8_H_15_NO_2_	2.02	1	Unknown	0004827	3		4.1 × 10^−3^	20,000	5000
130.0651	14.29	C_9_H_7_N	1.80	1	Unknown	0004827	0	103.05 (100);	8.4 × 10^−4^	94,000	50,000
167.1067	15.12	C_10_H_14_O_2_	1.75	1	Perillic acid	0000070	3	149.008 (30) 121.028 (20) 93.07 (80) 79.05 (100)	3.4 × 10^−2^	5000	15,000
374.2526	12.53	C_19_H_35_NO_6_	1.72	1	Dodecanedioyl-L-carnitine	0013327	4	231.1585 (50); 130.05 (40);85.02 (35)	1 × 10^−3^	44,264	29,738
164.0367	12.51	C_5_H_9_NO_3_S	1.58	1	Unknown	0001890	5	122.0269 (100); 76.02 (70)	2 × 10^−2^	60,000	30,000
245.1595	1.04	C_8_H_18_N_4_O_2_ + [C_2_H_2_O]	1.55	1	Dimethylarginine acetlylated	0001539	3	203.14 (30); 158.08 (100); 115.08 (70); 70.06 (50)	2 × 10^−3^	5000	8000
146.0600	14.29	C_9_H_7_NO_2_-[O]	1.36	1	Indolecarboxylic acid	0002285	1	118.65 (100) 91.054 (70) 65.039 (60) 128.049 (30)	2.4 × 10^−2^	50,000	25,000

The main urinary biomarkers identified in rats after Golden Berry consumption. Observations include mass-to-charge ratio (*m*/*z*), retention time (Rt), chemical formula, Variable Importance in Projection (VIP) Score, charge state (Z), tentative identification, Human Metabolome Database (HMDB) reference, mass error in parts per million (ppm), MS2 measured peak intensities, *p*-value for significance, and average relative intensity before (B) and after (A) Golden Berry consumption. The table focuses on biomarkers related to metabolic health, highlighting Golden Berry’s influence on metabolic pathways and providing insights into its potential health benefits.

## Data Availability

Data are contained within the article.
